# Supplementary material for: Association between visceral fat and bone mineral density in perimenopausal women
Source: PeerJ. 2025 Feb 13;13:e18957. doi: 10.7717/peerj.18957 (PMC11830370; doi:10.7717/peerj.18957)
Supplement: Supplemental Information 2 [file peerj-13-18957-s002.docx]

assignment of independent variables and dependent variables

| variable | code |
| --- | --- |
| degree of education | >post-graduate =3, college =2, senior =1, <junior =0 (control group) |
| Drink milk | Yes =1, No =0 (control group) |
| Calcium intake | Yes =1, No =0 (control group) |
| day length | >30min=1, <30min=0 (control group) |
| Mode of delivery | Caesarean=1, Eutocia=0 |
| Menstrual condition | Menopause =1, not menopausal =0 (control group) |
| BMD1 | Low bone mass =1, Osteoporosis=2, Control =0, abnormal bone mass=1/2 |
